# “Agentic fuel” and “Social dividends”: a mixed-methods explanatory study of optimism as a mediator between gratitude, mindfulness, and psychological wellbeing among Chinese university EFL students

**DOI:** 10.3389/fpsyg.2025.1734667

**Published:** 2026-01-29

**Authors:** Wenjie Zhao

**Affiliations:** Department of Public Course Teaching, Henan Vocational University of Science and Technology, Zhoukou, Henan, China

**Keywords:** Chinese university students, gratitude, mindfulness, mixed-methods, optimism, psychological wellbeing, structural equation modeling (SEM)

## Abstract

**Introduction:**

This study explored how gratitude and mindfulness contribute to psychological wellbeing (PWB) among Chinese university students, using an explanatory sequential mixed-methods design to test a structural model and qualitatively examine the lived experiences behind the statistical pathways.

**Methods:**

In the quantitative phase, 481 EFL students (61.9% female, *M*_*age*_ = 20.34) completed validated measures of gratitude, mindfulness, optimism, and PWB, with data analyzed through Structural Equation Modeling (SEM). Subsequently, 18 participants from this sample were purposefully selected for semi-structured interviews, analyzed via reflexive thematic analysis.

**Results:**

The SEM results showed a good model fit, confirming optimism as a robust mediator. Gratitude (β = 0.36) and mindfulness (β = 0.29) significantly predicted optimism, which strongly predicted PWB (β = 0.60). Bootstrapping revealed that optimism fully mediated the mindfulness-PWB link (indirect β = 0.17, *p* < 0.001; direct β = 0.06, *p* = 0.244) but only partially mediated the gratitude-PWB link (indirect β = 0.22, *p* < 0.001; direct β = 0.14, *p* = 0.021). Qualitative findings enriched these results, portraying optimism as an “agentic fuel” for eudaimonic action, with gratitude fostering it through “evidentiary reappraisal” and mindfulness through “cognitive decoupling.” The direct effect of gratitude was attributed to its role as an immediate “in-the-moment social dividend,” enhancing positive relations within PWB.

**Discussion:**

These findings suggest that optimism is a key mechanism linking positive resources to wellbeing, though not exclusively. Mindfulness supports wellbeing by creating mental space for optimism, while gratitude operates dually, building optimism for future benefits and directly enhancing social-relational aspects of PWB in the present. Interventions should target these distinct mechanisms to optimize student wellbeing.

## 1 Introduction

Psychological wellbeing (PWB), a cornerstone of human flourishing, extends beyond the mere absence of psychopathology to encompass multidimensional positive functioning, including self-acceptance, purpose, positive relationships, autonomy, and personal growth (Keyes, 2005; Ryff and Singer, 1998). High PWB is crucial for fostering resilience, adaptive coping, and overall life satisfaction (Diener et al., 2010; Ryff and Keyes, 1995). This construct is particularly salient for university students, a population facing significant stress. The convergence of intense academic pressure, newfound autonomy, and complex social demands strains student mental health, highlighting the need to identify protective resources (Li and Hasson, 2020; Morales-Rodríguez et al., 2020).

Positive psychology has identified key resources that bolster wellbeing. Gratitude—recognizing the positive aspects of life and others' kindness (Emmons and McCullough, 2003)—is consistently linked to higher life satisfaction, lower depression, and enhanced social support in students (Deichert et al., 2021; Kardas et al., 2019). Similarly, mindfulness—the intentional, non-judgmental awareness of the present moment (Kabat-Zinn, 2003)—is a powerful tool for reducing student stress, anxiety, and improving emotion regulation (Hofmann et al., 2010; Pawlak et al., 2025; Rehman et al., 2023; Zhang and Fathi, 2024).

However, treating these variables as isolated predictors overlooks the specific cognitive architecture required to translate positive affect into sustained wellbeing. While gratitude and mindfulness improve current states, PWB is inherently future-oriented, requiring goal-directed striving and growth. We propose that the essential link—and the primary mediator in this study—is optimism: the generalized positive expectancy for one's future (Scheier and Carver, 1985). Theoretically, we conceptualize optimism not as a parallel trait, but as a cumulative cognitive consequence of these practices. Drawing on the Broaden-and-Build theory (Fredrickson, 2004) and Self-Regulation Theory (Scheier and Carver, 1985), gratitude and mindfulness function as the “builders” of cognitive resources, while optimism represents the resulting structure.

Specifically, gratitude provides the “evidentiary basis” for optimism by focusing attention on past support and present blessings (Emmons and McCullough, 2003), thereby constructing a rational basis for expecting future success. Simultaneously, mindfulness fosters optimism by “decoupling” individuals from ruminative loops and negativity bias (MacDonald and Baxter, 2017), creating the necessary mental clarity for positive expectancies to emerge. Once established, this optimistic outlook acts as the proximal “agentic fuel” that motivates the difficult behaviors defining eudaimonic wellbeing (Ryff and Keyes, 1995).

This mechanistic approach addresses a critical gap in the literature regarding cultural specificity. While Western studies have established simple associations between these variables, few have examined how they function within the high-stakes, culturally distinct environment of Chinese higher education. Chinese EFL students navigate a unique “double bind”: the traditional Confucian emphasis on social harmony and collective obligation, juxtaposed with the intense, individualistic competition of the modern academic market (often termed “involution” or neijuan) (Ma et al., 2017). In this specific milieu, we argue that the function of gratitude extends beyond Western models of personal mood enhancement to serve as a vital “social dividend” that buffers against competitive isolation. By formally modeling how these distinct cultural and cognitive pathways converge to build optimism, this study moves beyond simple replication to offer a novel, culturally situated explanatory model of student flourishing.

The present study employed an explanatory sequential mixed-methods design to address these gaps. We aimed to: (1) quantitatively test a structural mediation model where optimism mediates the relationship between gratitude, mindfulness (predictors), and psychological wellbeing (outcome) among Chinese university students, and (2) qualitatively explore the lived experiences that explain how and why these pathways function.

## 2 Literature review

### 2.1 Core elements of wellbeing

Psychological wellbeing (PWB) is a multidimensional construct central to human flourishing, extending beyond the mere absence of psychopathology (Ryff and Singer, 1998). Grounded in positive psychology, optimal functioning includes the presence of positive emotions (Fredrickson, 2001; Keyes, 2005), a sense of autonomy and self-determination (Ryan and Deci, 2000), and the cultivation of strong social connections (Diener and Seligman, 2002; Keyes, 1998). Crucially, it also involves deeper cognitive components such as self-acceptance and a sense of purpose (Steger et al., 2006, 2009). While the foundational six-factor model (Ryff and Keyes, 1995) remains widely used, recent network analyses suggest PWB functions as an interconnected system where self-acceptance often serves as the central node linking environmental mastery and purpose (Blasco-Belled and Alsinet, 2022).

The importance of PWB is well-documented; it fosters life satisfaction (Diener et al., 2010), serves as a protective factor against mental disorders (Keyes, 2007), and buffers against the detrimental effects of stress (Fredrickson, 2001). Positive emotions broaden cognitive and behavioral repertoires, building enduring resources over time (Fredrickson, 2004; Fredrickson and Joiner, 2002). Conversely, deficits such as perceived social isolation pose significant risks for mental health (Cacioppo and Hawkley, 2009). This interplay of risks and resources is particularly relevant for university students, who face a complex convergence of academic pressure and social demands (Morales-Rodríguez et al., 2020; Vázquez et al., 2009).

Research on student populations identifies key internal and behavioral predictors of wellbeing. Cognitively, self-efficacy (Siddiqui, 2015) and academic passion (Vallerand, 2012) are foundational. Structural equation models highlight meaningfulness as a strong predictor of satisfaction, interacting with resources like mindfulness (Crego et al., 2020). Behaviorally, stress management is paramount (Li and Hasson, 2020); adaptive coping strategies enhance PWB, whereas maladaptive strategies increase distress (Meng and D'Arcy, 2016). Resilience, therefore, is an active adaptation process predicting autonomy and growth (Klainin-Yobas et al., 2021), supported by healthy lifestyle choices (Ozpolat et al., 2012).

Collectively, the literature depicts student wellbeing as a multifaceted construct (Morales-Rodríguez et al., 2020) influenced by a web of interconnected cognitive factors, emotional resources, and behavioral patterns. While identifying individual predictors is useful, a significant gap remains in understanding how foundational resources like gratitude and mindfulness interact to cultivate wellbeing. A clearer understanding of these mechanisms is required to create more holistic interventions for student populations.

### 2.2 Gratitude and mental health

Gratitude, a fundamental human emotion, involves a profound recognition of the positive aspects of one's life and an appreciation for the kindness of others (Emmons and McCullough, 2003). The association between gratitude and enhanced psychological wellbeing is extensively documented. Individuals who regularly engage in grateful behaviors exhibit lower levels of depression and anxiety, alongside higher levels of life satisfaction (Emmons and McCullough, 2003; Watkins et al., 2003). This influence extends beyond mental health, as practicing gratitude has also been linked to improved physical health outcomes, such as better sleep quality and enhanced immune function (Emmons and Stern, 2013).

Beyond its direct benefits, gratitude's power appears to lie in the multifaceted mechanisms through which it operates, which can be broadly understood through two key pathways: interpersonal and intra-psychic. First, gratitude is an inherently social and interpersonal emotion. Theoretical models describe gratitude as functioning through a “find-remind-and-bind” mechanism (Algoe et al., 2008). It helps individuals find new social partners, reminds them of the value of existing relationships, and binds them closer to others through reciprocal kindness. Expressing gratitude not only strengthens existing relationships but also cultivates a sense of belonging and reciprocity within communities (Algoe et al., 2008). This social function is a critical mechanism for wellbeing. For example, gratitude is known to enhance the positive effects of social support (Deichert et al., 2021), and structural equation models confirm that it has an indirect effect on wellbeing by improving social support (Lin and Yeh, 2014). This social-relational pathway appears particularly salient in collectivist cultural contexts; among Chinese university students, gratitude is a strong predictor of prosocial tendencies, which in turn mediate the path to PWB (Man and Jing, 2025). This social function also extends to conflict resolution, facilitating forgiveness and understanding (McCullough et al., 1998). Given that cultural norms shape how gratitude is expressed and received (Ma et al., 2017; Tsang, 2006), understanding this social component is vital.

Second, in addition to its social function, gratitude's influence operates through a range of intra-psychic and cognitive pathways, predicting wellbeing independently of core personality traits (Lin, 2015; Wood et al., 2009). Cognitively, gratitude acts as a filter that reorients attention. It encourages a “positive cognitive bias,” where individuals habitually scan their environment for beneficial inputs rather than threats (Wood et al., 2010). A primary outcome of this mechanism is its link to coping and resilience. Grateful individuals are more likely to utilize active coping styles rather than avoidance (Lin and Yeh, 2014). This cultivation of gratitude is deeply intertwined with resilience, with structural models showing both are strong, direct predictors of positive outcomes (Arnout and Almoied, 2021). This link suggests that gratitude interventions are a valuable strategy for promoting resilience, even during large-scale stressors like the COVID-19 pandemic (Geier and Morris, 2022). Gratitude also functions by altering other internal states. For example, it has been shown to be a key mediator, partially explaining the positive relationship between emotional intelligence and subjective wellbeing (Geng, 2018), and it also fosters self-esteem (Hemarajarajeswari and Gupta, 2021; Lin, 2015). Recent research with Chinese college students has clarified this cognitive function further, showing that gratitude works in concert with positive reappraisal (a coping strategy) to predict wellbeing, largely by cultivating an internal “peace of mind” (Du and Liu, 2025).

However, we selected optimism as the primary mediator over these other established mechanisms (e.g., social support, resilience, coping) because of its unique role as a proximal cognitive antecedent to action. While social support is an external resource, and resilience and coping are often behavioral outcomes of a positive mindset, optimism represents the generative cognitive stance—the “expectancy of success”—that makes those behaviors possible (Carver and Scheier, 2014). Eudaimonic wellbeing (PWB) requires agentic striving toward purpose and growth; individuals are unlikely to engage in such striving (coping) or reach out for help (social support) unless they first possess the internal belief that positive outcomes are attainable. Therefore, while gratitude may indeed foster peace of mind or social connection, we posit that its translation into active PWB depends primarily on its ability to build this generalized positive expectancy (optimism). Optimism thus serves as the necessary “cognitive bridge,” transforming the retrospective appreciation of gratitude into the prospective motivation required for psychological functioning.

### 2.3 Mindfulness and student wellbeing

Mindfulness, rooted in Eastern contemplative traditions, is broadly defined as the intentional and non-judgmental awareness of present-moment experiences (Kabat-Zinn, 2003; Bishop et al., 2004). Research has consistently demonstrated its benefits for mental health, showing that mindfulness-based interventions are effective for reducing symptoms of anxiety, depression, and stress (Hofmann et al., 2010). The practice is thought to facilitate emotion regulation (Keng et al., 2013) and improve cognitive processes like attention and executive functioning (Chiesa et al., 2011; Fathi et al., 2023), primarily by fostering cognitive flexibility and helping individuals disengage from automatic, habitual patterns of thinking (Lutz et al., 2008). While mindfulness is often an individual-level practice, it also holds promise for enhancing interpersonal relationships by fostering empathy, compassion, and prosocial behavior (Brown et al., 2009; Klimecki et al., 2014).

A growing body of research highlights the potential of mindfulness to enhance psychological wellbeing specifically among university students. Structural equation modeling, for example, has been used to confirm a direct, positive predictive effect of mindfulness on PWB in this population (Klainin-Yobas et al., 2016), with similar positive outcomes reported for high-stress groups like medical students (Sampath et al., 2019). Beyond this direct link, research is actively exploring the specific intra-psychic mechanisms driving this benefit. A primary mechanism appears to be the reduction of negative cognitive patterns. Mindfulness is thought to contribute to wellbeing by fostering present-moment awareness, which directly reduces rumination (MacDonald and Baxter, 2017). More complex models support this, suggesting a specific serial pathway where mindfulness enhances psychological flexibility, which in turn reduces rumination, thereby improving mental health (Feng et al., 2025). This cognitive shift also relates to problem-solving, as mindfulness may allow students to approach challenges with more constructive, solution-focused thinking (Arslan and Asici, 2022).

However, to fully understand how mindfulness translates into eudaimonic wellbeing (e.g., purpose, growth), it is necessary to identify the motivational mechanism that links “awareness” to “action.” We posit that optimism functions as this key mediator. Theoretically, mindfulness facilitates optimism through the mechanism of “reperceiving” or “decentering” (Shapiro et al., 2006). By training individuals to observe their thoughts non-judgmentally, mindfulness helps them disengage from the automatic catastrophic thinking and negativity bias that typically fuel pessimism. This “cognitive decoupling” clears the mental space for more positive, adaptive expectancies to emerge. While mindfulness provides the regulatory capacity to stop negative spirals, optimism provides the generative belief that future outcomes will be favorable. Thus, we propose that optimism acts as the essential explanatory mechanism: mindfulness reduces the cognitive barriers to hope, allowing optimism to flourish, which in turn fuels the active engagement required for psychological wellbeing.

In conclusion, mindfulness emerges as a promising tool for promoting psychological wellbeing in university students through multiple, complex pathways. Despite this, it is important to note that the precise conceptualization and measurement of mindfulness remain subjects of scientific debate (Grossman et al., 2004), and variability in interventions complicates the generalizability of results (Van Dam et al., 2018). By positioning optimism as a distinct mediator, this study seeks to clarify how the self-regulatory benefits of mindfulness are translated into the motivational outlook necessary for student flourishing.

### 2.4 The Influence of optimism on mental health

Optimism, characterized as a general expectation of positive future outcomes (Scheier and Carver, 1992), is a foundational construct in positive psychology (Seligman and Csikszentmihalyi, 2000). Its influence is broad, linking to superior physical and psychological health. Physiologically, an optimistic outlook is associated with enhanced immune functioning and reduced cardiovascular risk (Boehm et al., 2012; Tindle et al., 2009). Behaviorally, optimists engage more frequently in health-promoting habits and report higher quality of life (Conversano et al., 2010; Rasmussen et al., 2009). Psychologically, the benefits are robust, with optimism consistently predicting greater emotional wellbeing and life satisfaction (Bouchard et al., 2017; Scheier et al., 2001).

A primary function of optimism is its role in fostering psychological resilience. Optimistic individuals navigate adversity more effectively, demonstrating greater psychological flexibility and a persistence in goal pursuit (Carver and Scheier, 2014). This is largely achieved through more adaptive coping strategies; optimists are more likely to reframe stressful situations in a positive light, use problem-solving strategies, and seek out social support rather than resorting to avoidance (Conversano et al., 2010). This adaptive approach serves as a critical buffer, fostering emotional regulation and mitigating the detrimental effects of negative emotions during times of stress (Carver et al., 2010). Beyond its role as a direct predictor, optimism also functions as a key *mediator*, translating other positive cognitive and emotional resources into wellbeing. Structural equation models have shown that optimism is a pathway through which other traits operate. For example, optimism partially mediates the relationship between a positive attributional style and subjective wellbeing (Zhang et al., 2014). It also serves a similar function for more existential constructs, partially mediating the link between having a sense of meaning in life and multidimensional wellbeing (Ho et al., 2010). This suggests that one's explanatory style or sense of purpose may build wellbeing precisely *because* they first cultivate an optimistic outlook.

This positive orientation extends to interpersonal functioning. Optimism is linked to enhanced social competence (Smith et al., 2008), forgiveness (McCullough and Laurenceau 2000), and the active building of social support networks (Bouchard et al., 2017). Furthermore, studies indicate that optimism and perceived emotional intelligence independently predict psychological wellbeing (Augusto-Landa et al., 2011). However, the construct is nuanced; prospective research suggests that while ‘positive expectations' buffer against depression, a ‘sense of invulnerability' may be more relevant for buffering anxiety (Kleiman et al., 2017). Additionally, excessive or unrealistic optimism can become maladaptive (Shepperd et al., 1996), and its expression is shaped by socio-cultural factors (Chang et al., 2013).

This body of research is particularly relevant for university students, whose cognitive resources are frequently tested. The direct link between optimism and subjective wellbeing is well-supported in student samples (Rand et al., 2020). In this context, optimism serves a critical mediating function, linking perceived stress and resilience to psychological wellbeing (Zaheer and Khan, 2022). It also appears to be the pathway through which gratitude and hope are combined to foster wellbeing (Kardas et al., 2019), likely by enhancing students' beliefs in their ability to manage challenges (Sabouripour et al., 2021). Therefore, optimism stands out as a significant factor in student wellbeing, influencing it directly and serving as a key pathway for other protective resources.

### 2.5 The present study

While the independent contributions of gratitude, mindfulness, and optimism to psychological wellbeing are well-established, there is a significant gap in understanding the specific mechanisms and interrelationships through which these constructs operate, particularly within non-Western populations. The existing literature, largely dominated by Western samples, often treats these variables in isolation or in simple bivariate relationships. Few studies have modeled the specific mediational pathway in which gratitude and mindfulness act as antecedents to optimism, and optimism, in turn, functions as the proximal mechanism for enhanced psychological wellbeing.

The selection of optimism as the primary mediator in this study is grounded in Self-Regulation Theory (Scheier and Carver, 1985), which posits that human behavior is driven by the expectancy of future outcomes. While gratitude and mindfulness improve current affective states, eudaimonic wellbeing requires goal-directed action (e.g., personal growth, mastery). We propose that gratitude and mindfulness act as the cognitive inputs that shape these expectancies. Gratitude provides an evidentiary basis for positive expectations by highlighting past success and support (Kardas et al., 2019), effectively training the mind to detect resource availability. Simultaneously, mindfulness creates the cognitive conditions for optimism by reducing the negativity bias and future-oriented anxiety that typically inhibit positive expectancies (Keng et al., 2013). Thus, we posit that optimism is the necessary cognitive bridge: gratitude and mindfulness build the belief that “outcomes will be good,” and that belief drives the engagement required for psychological wellbeing.

This gap is particularly salient in the context of Chinese university students. This population faces a unique and well-documented confluence of intense academic pressure, collectivistic social expectations, and rapid cultural change, all of which place significant strain on psychological wellbeing (e.g., Tang et al., 2019). Understanding the interplay of indigenous (e.g., culturally-inflected gratitude) and cultivated (e.g., mindfulness) resources in this high-stress environment is of paramount importance.

Finally, quantitative models, while powerful for testing pathways, cannot capture the *lived experience* behind these relationships. We do not know *how* Chinese students cognitively or affectively translate a feeling of gratitude into a future-oriented expectation, or *why* a mindful, non-judgmental stance fosters optimism. A mixed-methods approach is therefore essential to provide a “thick description” (Geertz, 1973) that illuminates the *phenomenological processes* underlying the statistical model.

This study aimed to address these gaps by employing an explanatory sequential mixed-methods design to (1) quantitatively test a structural mediation model where optimism mediates the relationship between gratitude, mindfulness (predictors), and psychological wellbeing (outcome) among Chinese university students, and (2) qualitatively explore the lived experiences that explain *how* and *why* these pathways function.

Based on the theoretical and empirical literature reviewed, we hypothesized:

**H1:** Gratitude will be a significant, positive predictor of optimism.
**H2:** Mindfulness will be a significant, positive predictor of optimism.
**H3:** Optimism will be a significant, positive predictor of psychological wellbeing, controlling for the effects of gratitude and mindfulness.
**H4:** Optimism will significantly mediate the positive relationship between gratitude and psychological wellbeing (an indirect effect).
**H5:** Optimism will significantly mediate the positive relationship between mindfulness and psychological wellbeing (an indirect effect).

## 3 Method

We employed an explanatory sequential mixed-methods design (Creswell and Plano Clark, 2018). This two-phase approach was selected to achieve both statistical generalization from a large sample and deep, contextual understanding from a subsample. The study was structured with a primary priority on the quantitative phase (QUAN → qual). The first, quantitative phase involved collecting and analyzing cross-sectional survey data from a large sample of Chinese university students. This phase was designed to test the hypothesized mediation model (Gratitude/Mindfulness → Optimism → Wellbeing) using Structural Equation Modeling (SEM). The second, qualitative phase utilized semi-structured interviews with a purposefully selected subsample of participants from the quantitative phase. This phase was designed to explain, elaborate, and illustrate the statistical relationships identified in the quantitative model. Specifically, it aimed to provide a rich, phenomenological account of the lived experiences of how optimism functions as a cognitive-affective mechanism linking the predictors to wellbeing within the participants' specific cultural and academic context.

### 3.1 Participants

The initial sample was drawn from undergraduate EFL (English as a Foreign Language) students at two large, comprehensive public universities in Eastern China (Shanghai and Nanjing). These sites were selected for their large, diverse student bodies and geographic representation of urban Eastern China, enhancing the potential generalizability of the findings to this population. An a priori power analysis using G^*^Power (Faul et al., 2007) indicated that our final sample (*N* = 481) was more than adequate to detect small-to-medium effects (*f*^2^ = 0.05) in our structural model, providing excellent statistical power (>0.95) and ensuring model stability. A total of 520 students were initially recruited for the quantitative phase. Data were systematically screened prior to analysis. Responses with significant missing data (i.e., >10% of items incomplete) were removed (*n* = 28). We also excluded participants who failed two or more embedded attention-check items (*n* = 4). Multivariate outliers were then identified using Mahalanobis distance (*p* < 0.001) and excluded (*n* = 11). The final, valid sample for the quantitative analysis consisted of 481 participants (*N* = 481). The mean age of this sample was 20.34 years (SD = 1.89, range = 18-24). The cohort included 298 participants identifying as female (61.9%), 181 as male (37.6%), and 2 (0.4%) who preferred not to specify gender. Participants represented a diverse range of academic disciplines, including humanities (30.1%), social sciences (28.5%), STEM (35.1%), and other fields (6.3%), enrolled in compulsory College English courses.

From the 481 participants in the quantitative sample, we purposefully selected 18 individuals for the qualitative follow-up. Participants were recruited from the pool of students who indicated willingness to be interviewed (*n* = 134). To ensure the qualitative data could adequately explain the statistical model, we applied a maximum variation sampling strategy (Patton, 2015) based on participants' standardized Z-scores. Selection was guided by three specific criteria:

1. Congruent High/Low Profiles (*n* = 10): We selected students whose scores were consistently in the top or bottom quartiles across all three variables (Gratitude/Mindfulness → Optimism → PWB). This allowed us to examine how the “ideal” model functions for flourishing students and how the mechanism collapses for those with low wellbeing. 2. Discordant/Deviant Cases (*n* = 8): To understand the nuances of the mediation, we specifically targeted “outliers” whose scores contradicted the main model—for example, students with high Mindfulness scores (> +1 SD) but low Optimism (< -1 SD). These cases were crucial for identifying barriers to the translation of positive resources into optimism. 3. Demographic Balance: Within these psychometric profiles, we balanced selection to ensure representation across gender (10 females, 8 males) and academic disciplines (STEM and Humanities) to prevent faculty-specific biases.

This subsample consisted of 10 females and 8 males (Mage = 21.1 years). Theoretical saturation of themes was reached after approximately 16 interviews, with two additional interviews conducted to confirm that no new major themes were emerging.

### 3.2 Data Collection Procedure

Following approval from the Institutional Review Board, participants were recruited through university course portals and campus advertisements. They were offered either course credit (for psychology courses) or a small monetary compensation (¥20 RMB) for their participation. Data were collected using a secure online survey platform (Wenjuanxing) in a single, 25 mins session. To ensure data quality, we embedded two attention-check items (e.g., “Please select “strongly agree” for this item”) within the survey. Prior to beginning, all participants provided digital informed consent, were assured of their anonymity and confidentiality, and were informed of their right to withdraw at any time. The survey was administered entirely in Mandarin Chinese.

The 18 selected participants were contacted via email and invited for a follow-up interview. All agreed to participate. The interviews were conducted in Mandarin by the first author, who is fluent in the language and trained in qualitative interviewing techniques. Interviews were conducted via secure video conferencing (Tencent VooV Meeting) to accommodate participants' schedules and lasted between 45 and 60 mins. Before each interview, the researcher reiterated the study's goals, reviewed confidentiality protocols, and built rapport. All interviews were audio-recorded and transcribed verbatim by a professional transcription service, and the transcripts were then checked against the audio for accuracy by a bilingual research assistant. All identifying information was anonymized in the transcripts.

### 3.3 Measures and materials

#### 3.3.1 Control variables

To isolate the unique effects of gratitude and mindfulness, we included gender, age, and socioeconomic status (SES) as covariates. The inclusion of these controls is grounded in empirical evidence demonstrating their potential to confound relationships with wellbeing (Berdida et al., 2023; Klainin-Yobas et al., 2021). Socioeconomic status acts as a structural resource that can buffer against stress and facilitate environmental mastery (Diener et al., 2018). Similarly, gender differences are frequently observed in self-reports of emotional functioning and social connectedness (Morales-Rodríguez et al., 2020), while age—even within university cohorts—correlates with developmental trajectories in dimensions such as environmental mastery and autonomy (Ryff and Singer, 1998). Controlling for these demographic factors ensures that the observed structural pathways reflect the distinct cognitive mechanisms of the predictors rather than background variance. Gender and age were self-reported. SES was measured using the 4-item Family Affluence Scale (FAS-II; Bøe et al., 2012), which assesses material assets (e.g., “Does your family own a car, van or truck?,” “Do you have your own bedroom for yourself?”) and has been validated in adolescent and young adult populations.

#### 3.3.2 Quantitative measures

All instruments, originally developed in English, were translated into Mandarin Chinese. A rigorous translation and back-translation procedure (Brislin, 1970) was conducted by a committee of three bilingual experts (two in psychology, one in translation studies) to ensure conceptual and linguistic equivalence. Unless otherwise noted, all items were scored such that higher values indicated a greater level of the construct.

##### 3.3.2.1 Gratitude

Participants' sense of gratitude was measured using the 6-item Gratitude Questionnaire (GQ-6; McCullough et al., 2002). Participants rated statements (e.g., “I have so much in life to be thankful for”) on a 7-point Likert scale (1 = 'strongly disagree' to 7 = 'strongly agree'). The scale demonstrated excellent internal consistency in the present sample (Cronbach's α = 0.88).

##### 3.3.2.2 Mindfulness

Mindfulness was assessed using the 15-item Mindful Attention Awareness Scale (MAAS; Brown and Ryan, 2003). Participants rated their frequency of mindful states on a 6-point scale (1 = “almost always” to 6 = “almost never”). A sample item is, “I find myself listening to someone with one ear, doing something else at the same time.” As per the scale's scoring protocol, scores were averaged, with higher scores indicating greater mindfulness. The MAAS showed good reliability in our study (α = 0.85).

##### 3.3.2.3 Optimism

Optimism was evaluated using an eight-item scale developed by Scheier and Carver (1985). This scale measures generalized positive outcome expectancies (e.g., “In uncertain times, I usually expect the best”). Responses were recorded on a 7-point Likert scale (1 = “strongly disagree” to 7 = “strongly agree”). The scale demonstrated good internal consistency (α = 0.84).

##### 3.3.2.4 Psychological Wellbeing (PWB)

We utilized the 18-item condensed version of the Psychological Wellbeing Scale (Ryff and Keyes, 1995), which assesses six dimensions: autonomy, environmental mastery, personal growth, positive relations, purpose in life, and self-acceptance (3 items per dimension). Participants responded on a 7-point Likert scale (1 = ‘strongly agree' to 7 = “strongly disagree”). As lower scores on this scale indicated higher wellbeing, all 18 items were reverse-coded prior to analysis so that higher scores consistently indicated greater PWB. The composite scale demonstrated high internal consistency (α = 0.91), and all six subscales also showed acceptable reliability (αs ranged from.78 to.86). In our measurement model, PWB was operationalized as a second-order latent factor indicated by the six first-order dimensions.

#### 3.3.3 Qualitative interview protocol

A semi-structured interview guide was developed to explore participants' lived experiences regarding the study constructs. The guide was informed by the initial quantitative results, focusing on *how* participants perceived gratitude and mindfulness practices (or lack thereof) influencing their future outlook (optimism) and, subsequently, their overall sense of wellbeing. The guide was pilot-tested with two students (not in the final sample) to refine question wording and flow.

Sample probes included: “Can you describe a recent time when feeling grateful, or noticing things to be thankful for, changed your perspective on a stressful situation?” and “You scored high [or low] on mindfulness. How do you think your tendency to be ‘in the moment' affects your feelings about the future or your ability to cope with challenges?” Follow-up questions were used to probe for specific examples and perceived causal links (e.g., “How did that feeling of gratitude *lead to* that change in perspective?”).

### 3.4 Data analysis

We conducted quantitative analysis using SPSS (Version 28) for preliminaries and AMOS (Version 28) for the primary SEM analysis. We first calculated descriptive statistics, Cronbach's α, and Pearson's correlations. Little's MCAR test (χ^2^ (112) = 120.5, *p* = 0.28) indicated data were missing completely at random, justifying the use of Full Information Maximum Likelihood (FIML) estimation for all SEM procedures. While assumptions of linearity and multicollinearity were met (VIFs < 2.5), significant multivariate non-normality (Mardia's test, *p* < 0.001) prompted the use of 5,000 bootstrap samples to generate robust standard errors and confidence intervals.

To ensure a rigorous application of Structural Equation Modeling (SEM) rather than a path analysis of observed variables, we employed the item parceling technique (Little et al., 2002). This approach was selected to enhance model parsimony, improve the indicator-to-sample size ratio, and minimize the impact of item-specific measurement error. For the unidimensional constructs (Gratitude and Mindfulness), we created three balanced parcels per construct using the factorial algorithm (Little et al., 2002). For Optimism, although the Life Orientation Test-Revised (LOT-R) is sometimes debated as bi-dimensional (optimism vs. pessimism), we followed the recommendation of Scheier et al. (1994) and seminal validation studies in Chinese university students (e.g., Lai et al., 1998) which support treating it as a unidimensional continuum of generalized positive expectancy. Consequently, we utilized the factorial algorithm to generate three balanced parcels for the single latent Optimism factor, ensuring that both positively and negatively worded items were distributed across parcels to balance method variance. For the multidimensional Psychological Wellbeing (PWB) construct, the six theoretical dimensions (autonomy, environmental mastery, personal growth, positive relations, purpose in life, and self-acceptance) served as the observed indicators for the second-order latent factor.

We followed the two-step approach (Anderson and Gerbing, 1988). The CFA specified a four-factor measurement model. To rigorously test discriminant validity, we compared this hypothesized model to a rival three-factor model merging Optimism and PWB. A chi-square difference test confirmed our four-factor model was a significantly better fit. We then tested the structural model, specifying paths from Gratitude and Mindfulness to Optimism (mediator) and from all three constructs to PWB (outcome), with age, gender, and SES modeled as exogenous covariates predicting the endogenous latent factors (Optimism and PWB). Model fit was evaluated using standard criteria (χ^2^/df < 3.0; CFI/TLI ≥0.90; RMSEA/SRMR ≤ 0.08) (Hu and Bentler, 1999). We tested all indirect effects using 5,000 bias-corrected bootstrap samples (Preacher and Hayes, 2008).

For the qualitative phase, Mandarin transcripts were analyzed in NVivo 12 using reflexive thematic analysis (Braun and Clarke, 2006, 2019). The analysis followed the standard procedural phases from data familiarization to theme definition. To enhance trustworthiness (Lincoln and Guba, 1985), two researchers independently coded 25% of the data, achieving high inter-coder reliability (Cohen's κ = 0.89) after consensus discussions. This process was supplemented by researcher memoing for reflexivity and by member checking with three participants to validate the interpretations. Integration occurred at the interpretation stage using a narrative-weaving approach (Fetters et al., 2013). We systematically used the qualitative themes to explain and provide a “thick description” (Geertz, 1973) of the processes underlying the final SEM pathways, enabling a more comprehensive explanation than either method could offer alone.

## 4 Results

### 4.1 Quantitative results

#### 4.1.1 Preliminary analyses

Descriptive statistics, Cronbach's alpha coefficients, and Pearson's bivariate correlations for all primary study variables are presented in Table 1. As shown, all scales demonstrated good to excellent internal consistency, with Cronbach's alpha values ranging from 0.84 to 0.91. The correlation matrix revealed that all primary variables were significantly and positively inter-correlated in the hypothesized directions. Notably, both gratitude (*r* = 0.51, *p* < 0.001) and mindfulness (*r* = 0.44, *p* < 0.001) were strongly associated with the mediator, optimism. In turn, optimism showed a very strong positive correlation with psychological wellbeing (PWB) (*r* = 0.68, *p* < 0.001). The control variables (age, gender, and SES) showed only weak or non-significant correlations with the main latent constructs, supporting their use as covariates rather than primary predictors.

**Table 1:** Descriptive Statistics, Cronbach's Alphas, and Pearson's Correlation Matrix (*N* = 481).

**Variable**	***M***	***SD***	**1**	**2**	**3**	**4**	**5**	**6**	**7**
1. Age	20.34	1.89	-						
2. Gender (Female = 1)	0.62	0.49	0.04	-					
3. SES	3.11	0.82	0.09^*^	0.01	-				
4. Gratitude	5.21	1.10	0.05	0.08	0.11^*^	(0.88)			
5. Mindfulness	4.02	0.95	0.02	0.03	0.07	0.48^***^	(0.85)		
6. Optimism	5.05	1.14	0.06	0.04	0.13^**^	0.51^***^	0.44^***^	(0.84)	
7. PWB (Composite)	5.14	1.03	0.08^*^	0.06	0.15^**^	0.56^***^	0.49^***^	0.68^***^	(0.91)

PWB, Psychological Wellbeing; SES, Socioeconomic Status. Cronbach's alpha reliability coefficients are presented in bold on the diagonal. Scale ranges: Gratitude, Optimism, PWB (1–7); Mindfulness (1–6); SES (1–5). *p* < 0.05, ^**^p < 0.01, ^***^p < 0.001.

#### 4.1.2 Measurement model evaluation

We first tested the full measurement model using CFA to establish construct validity. This model specified four latent factors: Gratitude (indicated by 6 items), Mindfulness (indicated by 15 items), Optimism (indicated by 8 items), and a second-order latent factor of PWB (indicated by its six first-order sub-factors: autonomy, environmental mastery, personal growth, positive relations, purpose in life, and self-acceptance). The measurement model demonstrated a good fit to the data: χ^2^(1084) = 2245.72, *p* < 0.001; χ^2^/*df* = 2.07; *CFI* = 0.94; *TLI* = 0.93; *RMSEA* = 0.047 [90% CI (0.044,0.050)]; and *SRMR* = 0.051. Convergent validity was established, as all standardized factor loadings were robust and significant (*p* < 0.001), ranging from 0.64 to 0.90. Furthermore, the Average Variance Extracted (AVE) for each construct exceeded the 0.50 threshold (Gratitude = 0.61, Mindfulness = 0.57, Optimism = 0.60, PWB = 0.68), indicating that the latent variables explained more than 50% of the variance in their respective indicators. Discriminant validity was confirmed using the Fornell and Larcker (1981) criterion, as shown in Table 2. The square root of the AVE for each construct (bolded diagonal) was greater than its inter-construct correlation with any other factor. We further confirmed discriminant validity using the heterotrait-monotrait ratio of correlations (HTMT); all HTMT values were well below the conservative 0.85 threshold, with the highest being 0.79 (between Optimism and PWB). While this HTMT value approaches the 0.85 guideline, we argue this high correlation is theoretically expected given the nature of the constructs. To provide a stringent empirical test, we compared our hypothesized four-factor model (Gratitude, Mindfulness, Optimism, PWB) against the alternative three-factor model (Gratitude, Mindfulness, and a single Optimism-PWB factor). The alternative three-factor model demonstrated an exceptionally poor fit to the data (χ^2^(1092) = 8874.31, *p* < 0.001; χ^2^/*df* = 8.13; *CFI* = 0.71; *TLI* = 0.68; *RMSEA* = 0.112). A chi-square difference test confirmed that the hypothesized four-factor model was a significantly better fit (Δχ^2^(8) = 6628.59, *p* < 0.001). Taken together, these results confirm the construct validity and factorial distinctiveness of our measures, justifying the separation of optimism and PWB in the structural model.

**Table 2:** Discriminant validity (Fornell-Larcker Criterion) for latent variables.

**Construct**	**1**	**2**	**3**	**4**
1. Gratitude	(0.78)			
2. Mindfulness	0.53^***^	(0.75)		
3. Optimism	0.59^***^	0.51^***^	(0.77)	
4. PWB	0.63^***^	0.55^***^	0.74^***^	(0.82)

PWB, Psychological Wellbeing. Diagonal values (in bold) are the square root of the Average Variance Extracted (AVE). Off-diagonal values are the latent variable correlations from the CFA. ^***^p < 0.001.

#### 4.1.3 Structural model and hypothesis testing

**Figure 1:**
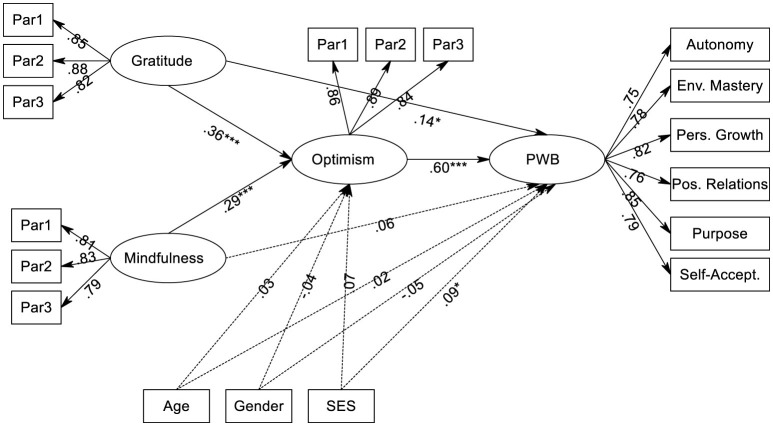
Final structural equation model of gratitude, mindfulness, optimism, and PWB (*N* = 481) Standardized path coefficients (β) are reported. Large ovals represent latent constructs; small rectangles represent observed indicators (item parcels for Gratitude, Mindfulness, and Optimism; subscale dimensions for PWB). Control variables (Age, Gender, and SES) are fully specified. Measurement errors are omitted for clarity. ^*^p < 0.05, ^***^p < 0.001.

After confirming the measurement model, we tested the full hypothesized structural model (see Figure 1). As shown in Figure 1, the visualized model displays the complete structural and measurement components, including the item parcels for the predictors and mediator, the six dimensions of PWB, and their respective standardized factor loadings. The control variables (age, gender, and SES) were modeled as distinct observed covariates predicting the endogenous latent factors. Results indicated that SES was a significant positive predictor of PWB (β = 0.09, *p* = 0.040), suggesting that higher socioeconomic status provides a small buffer for wellbeing. In contrast, neither age (β = 0.02, *p* = 0.642) nor gender (β = −0.05, *p* = 0.487) were significant predictors of PWB.

The structural model demonstrated a good fit to the data, identical to the measurement model: χ^2^(1112) = 2301.19, *p* < 0.001; χ^2^/*df* = 2.07; *CFI* = 0.94; *TLI* = 0.93; *RMSEA* = 0.047 [90% CI (0.044, 0.050)]; and *SRMR* = 0.053. The standardized path coefficients for the final model are presented in Figure 1. Both gratitude (β = 0.36, *p* < 0.001) and mindfulness (β = 0.29, *p* < 0.001) significantly and positively predicted optimism. Regarding the control variables, none were significant predictors of optimism. Their specific standardized coefficients are reported fully in Figure 1. In total, the model explained 39% of the variance in optimism (*R*^2^ = 0.39). As hypothesized, optimism was the strongest predictor of psychological wellbeing (β = 0.60, *p* < 0.001). A significant direct effect remained for gratitude (β = 0.14, *p* = 0.021), but the direct effect from mindfulness to PWB became non-significant (β = 0.06, *p* = 0.244). Among the covariates, SES was a small but significant predictor of PWB (β = 0.09, *p* = 0.040). This model accounted for a substantial 62% of the variance in psychological wellbeing (*R*^2^ = 0.62).

To formally test the hypothesized indirect effects of gratitude and mindfulness on PWB via optimism, we used the bias-corrected bootstrapping procedure (5,000 resamples). The results are presented in Table 3. • *Gratitude*→*Optimism*→*PWB:* The indirect effect was significant and positive [Standardized β_indirect_ = 0.22, 95% CI (0.16, 0.29), *p* < 0.001]. Because the direct effect (β = 0.14, *p* = 0.021) also remained significant, this finding supports partial mediation. • *Mindfulness*→*Optimism*→*PWB:* The indirect effect was also significant and positive [Standardized β_indirect_ = 0.17, 95% CI (0.11, 0.24), *p* < 0.001]. Because the direct effect (β = 0.06, *p* = 0.244) was non-significant, this finding supports full mediation. In summary, the quantitative results support our hypothesized model. Optimism was found to be a robust mediator, fully explaining the link between mindfulness and PWB, and partially explaining the link between gratitude and PWB.

**Table 3:** Bootstrapping results for direct, indirect, and total effects on Psychological Wellbeing (PWB).

**Effect**	**β**	**Boot SE**	**95% bias-corrected CI**	***p***
**Indirect effect (via Optimism)**				
Gratitude → Optimism → PWB	0.22	0.033	[.16,0.29]	< 0.001
Mindfulness → Optimism → PWB	0.17	0.031	[.11,0.24]	< 0.001
**Direct effect (on PWB)**				
Gratitude → PWB	0.14	0.061	[.02,0.26]	0.021
Mindfulness → PWB	0.06	0.050	[-0.04,0.16]	0.244
Optimism → PWB	0.60	0.052	[.49,0.70]	< 0.001
**Total effect (on PWB)**				
Gratitude → PWB	0.36	0.044	[.27,0.45]	< 0.001
Mindfulness → PWB	0.23	0.047	[.14,0.32]	< 0.001

β = Standardized coefficient; SE, Standard Error; CI, Confidence Interval. Bootstrapping based on 5,000 resamples. All effects are controlled for age, gender, and SES.

### 4.2 Qualitative results: explaining the model

To explain and elaborate upon the quantitative findings, we analyzed the semi-structured interviews from 18 purposefully selected participants. The reflexive thematic analysis yielded four key themes that directly map onto the pathways of the structural equation model. These themes explain *how* gratitude and mindfulness cultivate optimism and *why* this optimism translates into psychological wellbeing. Crucially, the analysis also provides a strong explanatory framework for the statistical difference observed in the mediation pathways—namely, why gratitude retained a direct effect on PWB while mindfulness did not.

The overarching finding is that participants did not experience these constructs as passive states, but as active cognitive processes. Optimism was not simply “hoping for the best”; it was an *active, reasoned expectation* built through specific cognitive reappraisals (fueled by gratitude) and cognitive decoupling (fueled by mindfulness). This optimism then served as the “agentic fuel” necessary to engage in the very behaviors that define eudaimonic wellbeing.

**Theme 1: Gratitude facilitates optimism through evidence-based reappraisal**

This theme directly explains the strong quantitative path from Gratitude to Optimism. Participants described gratitude not as a fleeting emotion, but as a deliberate cognitive act of *re-appraising their present situation*. This reappraisal provided tangible, concrete “evidence” that they could then use to challenge pessimistic projections about the future. Optimism, in this context, was the logical *conclusion* of this evidence-based review.

Jia, a high-gratitude/high-PWB participant, articulated this process when discussing her academic stress:

“It's easy to get overwhelmed and think, 'I'll never graduate, the job market is terrible.' But when I stop and think about what I'm grateful for... it's not abstract. It's 'I am grateful for my professor who *actually* helped me last week.' Or 'I'm grateful I passed that difficult statics exam.' It's like building a case file. This good stuff is real. So, logically, why should I expect *only* bad stuff in the future? It makes being optimistic feel more rational, less like... like a fantasy.” (Jia, 21, Female)

Another participant, Chen (high-gratitude/high-optimism), linked this to a form of “proof” that counteracted academic anxiety:

“My default is to worry. But when I force myself to notice [gratefulness], it's like... I have proof that I am supported. I have proof that I have succeeded before. This proof acts like an anchor. The future feels uncertain, like a storm, but I have this anchor of evidence. So I don't get washed away by 'what ifs.' I just feel... steady. I expect I'll be okay.” (Chen, 20, Male)

This theme illustrates that gratitude's path to optimism is not mystical, but cognitive. It is a process of evidentiary reappraisal where individuals use a structured review of present and past ‘positives' as a rational basis for generating positive future expectancies.

**Theme 2: Mindfulness enables optimism through cognitive decoupling**

This theme explains the quantitative path from Mindfulness to Optimism. In contrast to gratitude's *anchoring* function, participants described mindfulness as a process of cognitive decoupling. It provided a mental “space” that *disentangled* their present-moment failures or anxieties from their long-term future identity. This decoupling “cleared the slate,” neutralizing negative projections and allowing a more neutral-to-positive future outlook to emerge.

Li-Wei, a high-mindfulness/high-optimism participant, described this decoupling after receiving a poor midterm grade:

“My first thought was, 'I'm a failure. This ruins my GPA. I won't get into grad school.' The [mindfulness] practice... it's not about ignoring that thought. It's about seeing it as just... a thought. I can observe 'anxiety is present' or 'a feeling of failure is here.' It's not 'I *am* a failure.' That thought is not *me*. When you do that, the thought loses its power. It doesn't get to write the story for next semester. The future is just... open again.” (Li-Wei, 22, Female)

Another participant, Ming (high-mindfulness/low-gratitude), described it as “de-fanging” anxiety:

“I get stressed, my heart pounds before a presentation. Instead of spiraling into 'I'm going to bomb this,' mindfulness lets me just... sit with the pounding heart. That's all it is. It's a physical sensation. It's not a prophecy. By just letting it be, it stops being a signal of future doom. The future then just becomes... the future. It's not scary. It's just next.” (Ming, 20, Male)

This theme explains the mechanism for the full mediation found in the SEM. Mindfulness doesn't *build* optimism in the same way gratitude does; rather, it *protects* it by intercepting and neutralizing the pessimistic spirals that arise from present-moment distress. By decoupling “what is” from “what will be,” it preserves optimism.

**Theme 3: Optimism as a motivator for eudaimonic action and wellbeing**

This theme provides a powerful explanation for the robust path from Optimism to Psychological Wellbeing (PWB). For participants, optimism was not a passive state of “waiting for good things,” but the primary agentic fuel for *creating* good things. This directly links to the eudaimonic components of Ryff's PWB scale: participants who expected a positive outcome were more motivated to engage in the difficult behaviors necessary for Personal Growth, Environmental Mastery, and achieving a Purpose in Life.

Mei, a participant with high scores across all three variables, explained:

“If you truly believe you won't succeed, why would you even try? Why spend all night coding if you think the program will just fail? Because I generally believe... 'I can probably figure this out‘ [Optimism], I am willing to *start*. I am willing to sit with the frustration. That feeling of *doing* it, of finally solving the problem [Environmental Mastery]... that, to me, *is* wellbeing. It's not just happiness, it's... competence.” (Mei, 21, Female)

Another participant, Kai (high-optimism/high-PWB), connected this directly to goal-setting and purpose:

“I have this goal to start a social club... It's a huge amount of work. My friends say, ‘Why bother? It might fail.' But I just have this feeling that it will be worthwhile [Optimism]. That feeling is what gets me to... you know, book the rooms, make the posters, talk to the administration [Purpose in Life/Agency]. When I see the club actually *meeting*... I feel like I'm building something. I feel... alive. The optimism was the spark.” (Kai, 22, Male)

This theme moves beyond the cliché that “optimism feels good.” It demonstrates that optimism's primary role in eudaimonic wellbeing is *motivational*. It is the cognitive prerequisite for the goal-directed action and persistence that constitute a “purposeful” and “masterful” life.

**Theme 4: Gratitude's direct effect on wellbeing via enhanced social connection**

This final theme provides a compelling explanation for the quantitative “puzzle”: why Gratitude retained a direct path to PWB, while Mindfulness did not. The interviews revealed that gratitude, unlike mindfulness, has an inherently social and relational component. Participants described the *act* of feeling grateful as an immediate, “in-the-moment” booster of their felt connection to others. This directly maps onto the Positive Relations with Others subscale of PWB, providing a pathway to wellbeing that is *independent* of future-oriented optimism.

An, a participant with high-gratitude but moderate-optimism, exemplified this:

“I might be very worried about my exams next month [low optimism]. But right now, when my roommate brings me a cup of tea... and I feel that rush of ‘thank you'... in that exact moment, I feel... so connected to her. I feel less alone. That feeling of ‘not-aloneness' [Positive Relations] is its own kind of wellbeing, even if my future worries are still there.” (An, 20, Female)

In contrast, participants consistently described mindfulness as an *internal, individuated* practice. While it helped them manage their *own* mind, it did not, by itself, enhance their felt connection to others.

As Li-Wei (high-mindfulness) stated:

“Mindfulness helps me deal with *my* stress... It's very internal. It doesn't really have anything to do with my friends. It's about me and my thoughts.”

This theme explains the direct effect found in the SEM. Gratitude provides an immediate “social dividend” by reinforcing the Positive Relations dimension of PWB, regardless of its separate, parallel function of building optimism. Mindfulness, being an internal practice, lacks this direct

## 5 Discussion

This study employed an explanatory sequential mixed-methods design to elucidate the mechanisms linking gratitude and mindfulness to psychological wellbeing (PWB) among Chinese EFL university students. The quantitative results (*R*^2^ = 0.62) confirmed that optimism serves as a robust mediator, fully explaining the relationship between mindfulness and PWB, while only partially mediating the link between gratitude and PWB. Our qualitative findings contextualize these statistical pathways, characterizing optimism not as a passive state but as “agentic fuel” for action. Specifically, gratitude cultivates this fuel through “evidentiary reappraisal,” whereas mindfulness does so via “cognitive decoupling.” Gratitude's unique direct effect is attributed to an immediate “in-the-moment social dividend.”

The robust path from optimism to PWB (β = 0.60) warrants attention given the high latent variable correlation (*r* = 0.74, HTMT = 0.79), which might suggest conceptual redundancy. However, our mixed-methods findings defend the distinctness of these constructs. Empirically, the CFA confirmed that treating them as separate factors yielded a superior model fit. Qualitatively, participants described optimism not as the emotional state of “feeling good” (a component of PWB) but as the cognitive prerequisite for action. As Mei (21, Female) explained, the belief that “I can probably figure this out” provided the necessary “spark” to engage in the challenging tasks required for “Environmental Mastery.” This creates a direct, empirically-grounded mechanism for Ryff and Keyes' (1995) eudaimonic model: optimism acts as the causal “engine” driving “Personal Growth” and “Purpose in Life.” Without this “agentic fuel,” participants reported a behavioral paralysis that eroded wellbeing. Thus, the high correlation reflects a powerful causal dependency rather than a methodological artifact. This “optimism-as-agency” framework raises important questions regarding its interplay with self-efficacy and resilience. Future research should investigate whether optimism functions as an antecedent “why bother” that precedes the “I can” of self-efficacy (Sabouripour et al., 2021; Siddiqui, 2015), clarifying the specific cognitive sequence needed to unlock intrinsic motivation, competence, and autonomy (Ryan and Deci, 2000).

Given the importance of optimism as agentic fuel, the next logical question is how it is cultivated. Our model positioned gratitude and mindfulness as key antecedents, and our qualitative data illuminated the two distinct cognitive mechanisms responsible. First, the strong path from gratitude to optimism was explained by a novel cognitive process: “Gratitude as Evidentiary Reappraisal.” This aligns with existing research linking gratitude and optimism (Kardas et al., 2019; Emmons and McCullough, 2003). Participants like Jia (21, Female) described an active process of “building a case file” of past successes and present supports. This was not just a fleeting emotion; it was a rational process of using documented “proof” to challenge pessimistic future projections. This extends Fredrickson's (2004) “broaden-and-build” theory by specifying the *cognitive content* of the building process; gratitude broadens perspective by anchoring it in a “steady” base of evidence, which then allows for the building of a more rational, robust optimism. This suggests gratitude interventions (Geier and Morris, 2022) may be most effective not just by eliciting positive feelings, but by training this specific cognitive skill.

The significant path from mindfulness to optimism, while also consistent with the literature (Rehman et al., 2023; MacDonald and Baxter, 2017), operated through a functionally distinct mechanism. The qualitative findings uncovered “Cognitive Decoupling.” While gratitude *adds* a positive evidentiary base, mindfulness *removes* a negative one. Participants described mindfulness (Kabat-Zinn, 2003) as a practice of “de-fanging” anxiety (Ming, 20, Male) and seeing a failure as “just... a thought,” not an identity (“I am not a failure,” Li-Wei, 22, Female). This act of cognitive decoupling (Bishop et al., 2004) stops the ruminative spiral that projects a present-moment failure into a “prophecy of future doom.” In this way, mindfulness does not *create* optimism; it *creates the mental space* for optimism to emerge by neutralizing pessimism. This aligns perfectly with its theoretical role in reducing reactivity (Hofmann et al., 2010) and fostering acceptance (Arslan and Asici, 2022).

Perhaps the most novel and theoretically significant finding of this study was the divergence in these mediation pathways. Explaining this statistical “puzzle”—the full mediation for mindfulness vs. the partial mediation for gratitude—was a key achievement of our mixed-methods approach. The reason for gratitude's remaining direct path to PWB ($\beta = 0.14$) became clear in the interviews. Participants explained this through what we termed “The ‘In-the-Moment' Social Dividend.” As An (20, Female) described, feeling grateful for her roommate's cup of tea provided an immediate feeling of “not-aloneness,” which she explicitly identified as “its own kind of wellbeing,” even if her future worries remained. This finding directly maps onto the “Positive Relations with Others” subscale of Ryff's (1995) PWB measure. This suggests gratitude has a dual-pathway function: it builds future-oriented wellbeing *indirectly* by providing “evidentiary fuel” for optimism, and it builds present-moment wellbeing *directly* by reinforcing the social connections (Keyes, 1998; Diener and Seligman, 2002) that are themselves a core component of PWB. This strongly supports the work of Algoe et al. (2008) on the relationship-strengthening function of gratitude and Deichert et al. (2021) on its role in amplifying social support.

Conversely, the mindfulness-PWB link was fully mediated by optimism. This result is notable, as it diverges from literature suggesting mindfulness can directly foster interpersonal wellbeing (e.g., Rehman et al., 2023). Our qualitative data, however, resolve this apparent contradiction by highlighting that participants experienced mindfulness as a strictly internal, individuated practice. As Li-Wei explained, “It's very internal. It doesn't really have anything to do with my friends.” This internalization likely reflects two factors: our use of the MAAS, which prioritizes awareness over compassion (Brown and Ryan, 2003), and the specific high-stress context (Ma et al., 2017), where students adopt mindfulness instrumentally to manage academic anxiety. Consequently, while mindfulness effectively supports emotion regulation (Keng et al., 2013), it does not yield the direct “social dividend” found in gratitude. Its contribution to eudaimonic wellbeing is therefore indirect: by achieving “cognitive decoupling,” mindfulness clears the mental stage, allowing the “agentic fuel” of optimism to drive subsequent growth and mastery.

## 6 Conclusion

This study utilized a mixed-methods design to explain the mechanisms linking gratitude and mindfulness to the psychological wellbeing of Chinese university students. Quantitative results confirmed that optimism serves as a robust mediator, fully explaining the influence of mindfulness and partially mediating that of gratitude. Qualitatively, optimism emerged not as passive hope but as “agentic fuel” for eudaimonic action. This fuel is cultivated through two distinct pathways: gratitude provides “evidentiary reappraisal” (constructing a rational basis for positive expectancies), while mindfulness facilitates “cognitive decoupling” (neutralizing pessimistic spirals).

The partial mediation of gratitude is explained by its unique provision of an “in-the-moment social dividend,” which enhances the relational components of wellbeing independent of future outlooks. Ultimately, this study reframes gratitude and mindfulness as complementary but non-interchangeable resources. To flourish under academic pressure, students benefit from a dual approach: using mindfulness to clear the cognitive stage of pessimism, and gratitude to build the evidentiary case for their own success.

## 7 Implications

Our findings offer several important contributions, beginning with a refined theoretical understanding of the links between positive resources and wellbeing. This study empirically conceptualizes optimism as the cognitive engine for eudaimonic action. This moves beyond viewing optimism as a mere positive expectancy; our qualitative data position it as the “spark” that translates belief into behavior. It is the psychological mechanism that bridges the gap between feeling good and actively engaging in the behaviors of personal growth and mastery that define psychological wellbeing.

This central mechanism, in turn, is cultivated by distinct resources. We propose that gratitude operates via a unique dual-pathway model. On one hand, it provides the “evidentiary reappraisal” that builds this optimistic fuel. On the other, it offers a direct “social dividend” that immediately enhances the relational components of wellbeing. This dual function helps explain why gratitude is such a robust predictor of PWB, as it simultaneously services both future-oriented agency and present-moment connection.

Mindfulness, in contrast, appears to function differently in our model. In our findings, the primary role of mindfulness *appears* to be permissive rather than generative. By “cognitively decoupling” individuals from ruminative spirals, mindfulness may act as a crucial “ground-clearing” operation. It neutralizes the pessimism that can block goal pursuit, and in doing so, allows the “agentic fuel” of optimism to emerge. Its value is therefore *primarily* indirect, becoming fully realized only when the mental space it creates is filled by a positive, future-oriented stance. This interpretation aligns with the full mediation observed in our statistical model, though we acknowledge this finding must be interpreted cautiously, as the high multicollinearity between optimism and PWB may have suppressed a smaller, direct statistical effect.

This more nuanced understanding of the mechanisms has direct, actionable implications for student wellness programs, counselors, and educators. A one-size-fits-all approach to positive psychology is clearly insufficient. Our findings allow for a more prescriptive model. For example, a student presenting with intense loneliness might be guided toward a gratitude intervention, where the primary goal would be to activate the “social dividend” and immediately bolster their sense of positive relations. Conversely, a student stuck in debilitating rumination over a recent failure would likely benefit more from a mindfulness intervention, where the specific goal is “cognitive decoupling” to stop the pessimistic spiral.

Building on this, universities could move beyond offering isolated workshops to design a powerful, sequential intervention based on our model. Such a program might begin with a module focused on “clearing the stage” with mindfulness, teaching students to detach from academic stress and self-critical thoughts. This could then be followed by a module on “building the evidence” using gratitude, guiding students to actively catalog their past successes and present supports. A final, integrating module on “igniting the fuel” would explicitly connect these skills, framing optimism not as wishful thinking, but as the *rational outcome* of a clear mind and an evidence-based perspective—the very “agentic fuel” required to achieve their academic and personal goals.

## 8 Limitations and suggestions for future research

While this mixed-methods study offers a nuanced model, several limitations highlight avenues for future research. First, the cross-sectional design prevents definitive causal conclusions, despite qualitative support for the model's direction. For instance, higher wellbeing might foster gratitude. Longitudinal studies, perhaps timed around academic stressors, are needed to establish temporal precedence and model changes in variables over time.

Second, reliance on self-report measures, although robust, limits findings to participants' perceptions. This is particularly relevant for the “agentic fuel” concept. Future research should incorporate behavioral measures, such as persistence on experimental tasks (e.g., unsolvable anagrams) following relevant inductions, or use daily diary methods to capture real-time enactments of the “social dividend” (e.g., recording gratitude expressions and subsequent social connection ratings). Third, our findings are specific to high-achieving Chinese EFL university students. The impact of gratitude's “social dividend” might be particularly pronounced in this collectivistic context. Cross-cultural replication in more individualistic settings is crucial to test the generalizability of the model and the relative prominence of the “social dividend” vs. “agentic fuel” pathways.

Finally, the proposed cognitive mechanisms (“reappraisal” and “decoupling”) are inferred from correlational data. Rigorous testing requires experimental designs, such as a 2 x 2 factorial intervention study randomizing participants to gratitude, mindfulness, combined, or control conditions. This would allow for causal testing of the predicted unique effects on social connection, pessimism reduction, and optimism.

## Data Availability

The raw data supporting the conclusions of this article will be made available by the authors, without undue reservation.
